# Antimicrobial Vitrimers Synthesized from Dipentaerythritol Pentaacrylate and 2‑Hydroxy-3-phenoxypropyl Acrylate for LCD 3D Printing

**DOI:** 10.1021/acs.biomac.5c00577

**Published:** 2025-06-24

**Authors:** Vilte Sereikaite, Aukse Navaruckiene, Vita Raudoniene, Danguole Bridziuviene, Paulius Cerkauskas, Saulius Lileikis, Kastytis Pamakstys, Egidija Rainosalo, Anne-Sophie Schuller, Christelle Delaite, Jolita Ostrauskaite

**Affiliations:** † Department of Polymer Chemistry and Technology, 70309 Kaunas University of Technology, Radvilenu Rd. 19, LT-50254 Kaunas, Lithuania; ‡ Biodeterioration Research Laboratory, State Scientific Research Institute Nature Research Center, Akademijos St. 2, LT-08412 Vilnius, Lithuania; § JSC 3D Creative, Mokslininku St. 2, LT-08412 Vilnius, Lithuania; ∥ Institute of Environmental Engineering, Kaunas University of Technology, Gedimino St. 50, LT-44239 Kaunas, Lithuania; ⊥ 52903 Centria University of Applied Sciences, Talonpojankatu 2, FI-67100 Kokkola, Finland; # Laboratoire de Photochimie et d’Ingénierie Macromoléculaires - EA4567, Université de Haute Alsace, Université de Strasbourg, 3b rue Alfred Werner, 68093 Mulhouse Cedex, France

## Abstract

In this study, two monomers, dipentaerythritol pentaacrylate and 2-hydroxy-3-phenoxypropyl acrylate, were used to synthesize antimicrobial vitrimers with and without a transesterification catalyst and investigate their properties. The addition of the comonomer 2-hydroxy-3-phenoxypropyl acrylate to the photocurable resin reduced its viscosity and shrinkage but increased the gel point and reduced the brittleness and increased flexibility of the resulting polymers. All vitrimers exhibited self-welding and reprocessability properties and thermoresponsive shape memory, maintaining two permanent shapes. All vitrimers showed high antimicrobial activity against widely spread bacteria and fungal strains, including medically important ones. The resin, composed of dipentaerythritol pentaacrylate (1 mol) and 2-hydroxy-3-phenoxypropyl acrylate (10 mol), was applied to LCD 3D printing technology, and the Y-shaped connector was printed. In addition, the antimicrobial activity makes these vitrimers particularly important for use in areas with high microbial concentrations, such as medical facilities.

## Graphical abstract

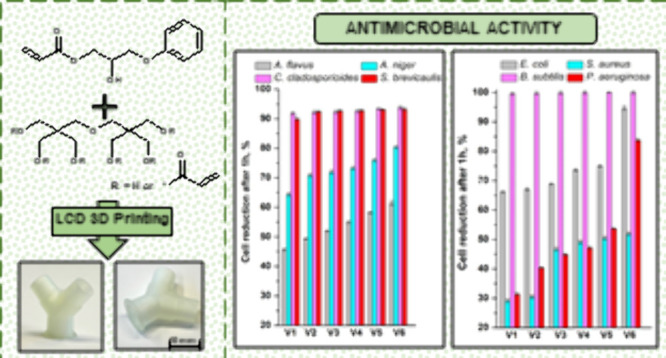

## 1 Introduction

In recent decades, plastics have been one of the most versatile materials, which have a variety of useful physical properties and are often resistant to moisture, biodegradation, and oxidation.^1^ Recently, a new class of renewable plastics known as vitrimers was presented to the scientific society.^2^ Vitrimers are cross-linked polymeric materials that can reorganize their topology through dynamic exchange reactions.^3^ Dynamic covalent bonds in their structure create strength and chemical resistance similar to those of thermosets, while allowing reprocessability under particular conditions similar to those of thermoplastics.^4,5^ The thermally activated transesterification reaction of ester and hydroxyl groups is one of the easiest implementations among bond exchange in adaptable networks.^6,7^ The most commonly used catalysts for vitrimer synthesis are strong bases, organic salts, and metal-based compounds that improve stability and production yield, as well as reprocessability of vitrimers.^8^ However, the majority of vitrimers depend on petroleum resources and catalysts are commonly toxic, leading to global environmental consequences.^9^ Novel biobased vitrimers were synthesized by thermal polymerization using tris­(2-aminoethyl)­amine and epoxidized methyl oleate with boric acid catalyst, and showed promising reprocessability results.^10^ Furthermore, lactic acid was mixed with epoxidized canola oil for the thermal synthesis of biobased vitrimers using zinc acetate catalyst.^11^ However, most biobased vitrimers are synthesized by thermal polymerization in the presence of a catalyst, and although catalyst-free vitrimers have already been reported in the literature, not many of them were synthesized from biobased materials.^6^ Bisphenol A diglycidyl ether was used in the thermal polymerization of catalyst-free epoxy vitrimers with self-healing abilities and fire retardancy.^12^ Tannic acid was used for thermal polymerization of fully biobased catalyst-free vitrimers with high mechanical strength and recyclability.^13^ Unfortunately, most of these vitrimers were synthesized by thermal polymerization, which is a time-consuming and energy-consuming process compared to photopolymerization.^14^ Photopolymerization can be performed at room temperature or even lower temperatures and starts rapidly after irradiation, fastening the vitrimer production time from hours to minutes.^15^ Biobased vitrimers were synthesized by photopolymerization of glycerol 1,3-diglycerolate diacrylate using Miramar A99 catalyst and showed weldability and reparability properties.^16^ Furthermore, photocured vitrimers based on glycerol and vanillin were synthesized using zinc acetylacetone hydrate transesterification catalyst and showed excellent self-healing, recyclability properties, and suitability for optical 3D printing.^17^ Although photopolymerization is superior to thermal polymerization with numerous advantages, there are only a few publications on catalyst-free biobased vitrimers prepared by photopolymerization. Recently, a biobased diamine dimethacrylate monomer derived from vanillin was used in photocurable vitrimer synthesis, which showed promising mechanical recyclability and suitability for DLP 3D printing technology. Although it is stated that these vitrimers were synthesized by photopolymerization, additional thermal postcuring was applied, leading to the possibility of thermal polymerization, suggesting that these vitrimers were not photocured, but dual-cured by the combination of photo and thermal polymerization.^18^ Because of that, in this work, attention was focused on the development of a novel catalyst-free photocurable vitrimer showing the possible way to synthesize it from biobased monomers, its comparison with vitrimers containing catalysts, and its application in optical 3D printing technology.

Optical 3D printing provides fast and accurate fabrication of products with low loss of materials and energy.^19^ It is a valuable technology in medical applications for the production of scaffolds, blood vessels, dental implants, and medical devices.^20,21^ However, medical tools and devices provide an ideal environment for the growth of microorganism and are one of the leading causes of hospital-acquired infections.^22^ Multidrug resistant microbes, such as *Staphylococcus aureus*, *Pseudomonas aeruginosa*, *Aspergillus terreus*, and others, have spread worldwide, increasing serious risk to human health.^23^ Due to that, antimicrobial activity is an enormous advantage in medical applications.^24^ Antimicrobial materials can prevent the growth of microbes and are widely used in medicine, textile industry, food packaging, coatings, etc.^25^

In this study, two monomers, dipentaerythritol pentaacrylate, which is possible to produce by chemical modification of vegetable oils, and 2-hydroxy-3-phenoxypropyl acrylate, which can be synthesized from a secondary product of the biodiesel production process, glycerol, were selected for the synthesis of new antimicrobial vitrimers.^26^ Photoinitiator ethyl­(2,4,6-trimethylbenzoyl) phenylphosphinate was chosen for this study due to its high reactivity and its ability to cure deep resin layers.^27^ The composition with the highest amount of 2-hydroxy-3-phenoxypropyl acrylate was selected for the investigation of vitrimeric properties because the addition of 2-hydroxy-3-phenoxypropyl increases the number of hydroxy groups in the vitrimer structure, which are crucial for transesterification reactions.^28^ Analogous compositions were prepared with the addition of a transesterification catalyst, zinc acetylacetonate hydrate, to compare the properties of catalyzed and noncatalyzed vitrimers. Zinc acetylacetonate hydrate was selected as it is one of the most widely used transesterification catalysts, which already showed promising results in vitrimer synthesis.^29^ A real-time photorheometer was used to find the most suitable composition for 3D printing. The thermal, mechanical, and antimicrobial properties of vitrimers were investigated and compared. Self-welding and reprocessability tests were performed to prove the recyclability of catalyst-free vitrimers and compare their properties with those of the vitrimer-containing catalyst. Furthermore, selected resin was applied in LCD 3D printing technology, which is among the most advanced 3D printing technology today and enables its application in a variety of areas including medicine, electronics, automotive, and others.

## 2 Materials

2-Hydroxy-3-phenoxypropyl acrylate (HPPA, Merck), dipentaerythritol pentaacrylate (DPEPA, Merck), zinc acetylacetonate hydrate (ZnAc, Merck), and ethyl­(2,4,6-trimethylbenzoyl) phenylphosphinate (TPOL, Fluorochem) were used as received (Figure 1). The compositions of the resins are listed in Table 1.

**Figure 1:**
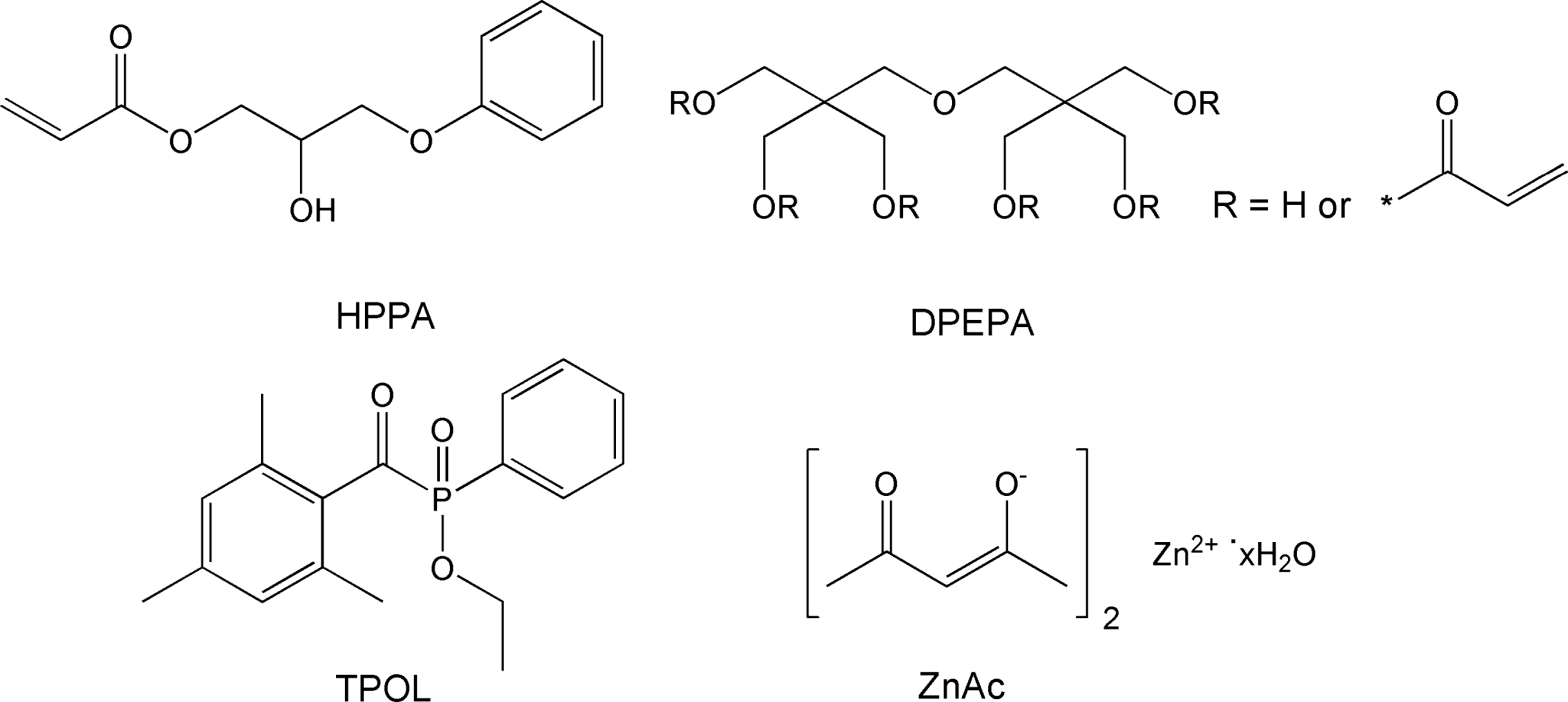
Chemical structure of 2-hydroxy-3-phenoxypropyl acrylate (HPPA), dipentaerythritol pentaacrylate (DPEPA), ethyl­(2,4,6-trimethylbenzoyl) phenylphosphinate (TPOL), and zinc acetylacetonate hydrate (ZnAc).

**Table 1:** Compositions of Resins **V1**–**V6**

resin	molar ratio of DPEPA and HPPA	amt of TPOL, mol %	amt of ZnAc, wt%
**V1**	1:0	3	
**V2**	1:10	3	
**V3**	1:30	3	
**V4**	1:90	3	
**V5**	0:1	3	
**V6**	1:90	3	5

Characterization techniques, experimental methodologies and FT-IR spectra are described in detail in the Supporting Information.

## 3 Results and Discussion

### 3.1 Photocuring Kinetics

The photocuring kinetics of the resins was investigated by real-time photorheometry. The results are presented in Table 2. The dependence of G’ of the resins on irradiation time is shown in Figure 2.

**Table 2:** Rheological Properties of Resins **V1**–**V6**

resin	storage modulus *G*′, MPa	loss modulus *G*′′, MPa	induction period, s	gel point *t*_gel_, s	shrinkage, %	viscosity η, Pa·s
**V1**	1.4·10^1^ ± 0.0	1.4 ± 0.0	0.0 ± 0.0	2.0 ± 0.0	1.6·10^1^ ± 0.0	7.7 ± 0.2
**V2**	2.4·10^1^ ± 0.1	3.7 ± 0.1	0.0 ± 0.0	2.0 ± 0.0	1.8·10^1^ ± 0.0	0.3 ± 0.0
**V3**	1.8·10^1^ ± 0.1	3.6 ± 0.1	0.0 ± 0.0	2.0 ± 0.0	1.5·10^1^ ± 0.0	0.3 ± 0.0
**V4**	1.4·10^1^ ± 0.0	3.6 ± 0.1	0.0 ± 0.0	6.8 ± 0.1	1.3·10^1^ ± 0.0	0.2 ± 0.0
**V5**	1.2·10^1^ ± 0.0	1.1 ± 0.0	0.0 ± 0.0	7.3 ± 0.1	1.2·10^1^ ± 0.0	0.2 ± 0.0
**V6**	1.4·10^1^ ± 0.0	3.8 ± 0.3	0.0 ± 0.0	1.1·10^1^ ± 0.0	1.2·10^1^ ± 0.1	0.3 ± 0.0

**Figure 2:**
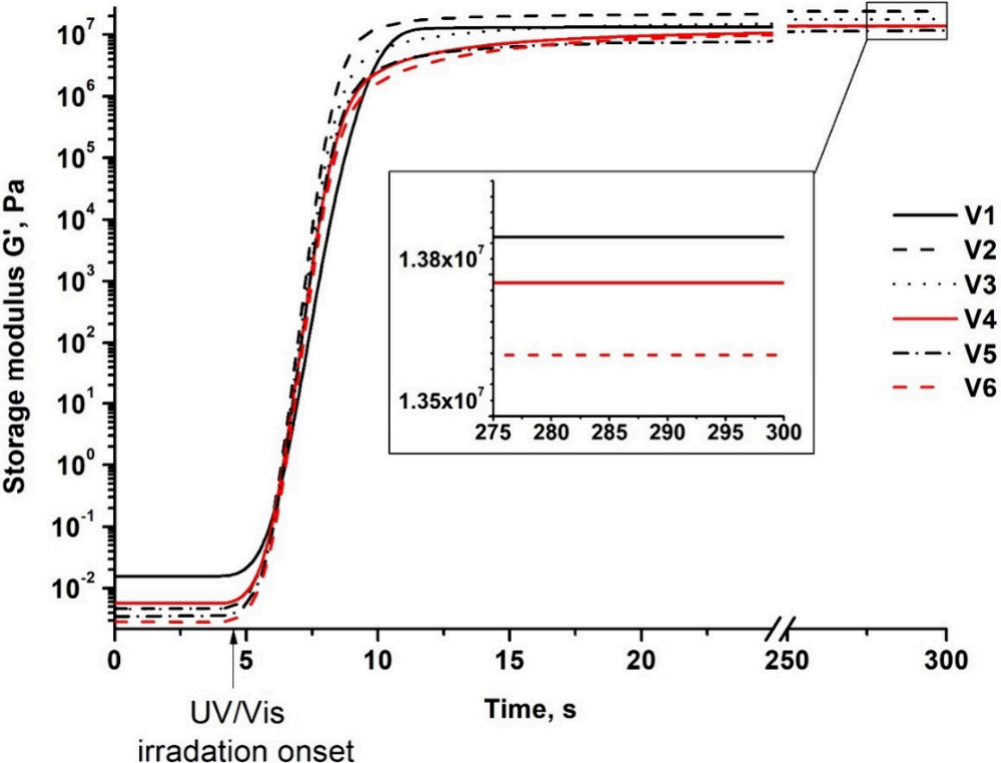
Dependence of the storage modulus of resins **V1**–**V6** on the UV/vis irradiation time.

The resins of pure monomers DPEPA (**V1**) and HPPA (**V5**), as well as resins prepared with different ratios of the two comonomers (**V2**–**V4** and **V6**), were investigated to evaluate the influence of the comonomer HPPA and its ratio on the photocuring kinetics. Polymer **V1** had a lower rigidity (described by the *G*′ value) compared to polymer **V2**, which was prepared with 1 mol of DPEPA and 10 mol of HPPA (1.4·10^1^ and 2.4·10^1^ MPa, respectively). This is because of the high number of acrylic groups of DPEPA that causes the fast formation of spatial hindrances that slow photopolymerization as unreacted acrylic groups cannot reach each other. The addition of HPPA increased the G’ value of the polymer because HPPA has only one acrylic group and aromatic structure, and it can easily overcome spatial hindrances formed by the DPEPA polymer network and react with unreacted acrylic groups of DPEPA.^30^ However, further addition of HPPA to the composition of resins greatly reduced the rigidity of polymers and the photocuring rate but also reduced the viscosity (η) and the shrinkage value. For example, when HPPA and DPEPA were changed from 1:10 (**V2**) to 1:90 (**V4**), the storage modulus was reduced from 2.4·10^1^ to 1.4·10^1^ MPa, the gel point was increased from 2.0 to 6.8 s and the shrinkage was reduced from 1.8·10^1^ to 1.3·10^1^ %. This is because of the formation of longer linear polymer chains between the cross-linking points by adding a higher amount of HPPA. This was confirmed by the results of the HPPA homopolymer **V5**, which was the least rigid and shrank less than the cross-linked polymers **V1** – **V4** and **V6**,^31^ as **V5** formed a linear polymer. The addition of ZnAc to the composition had no impact on the rigidity of the samples, but increased η from 0.2 (**V4**) to 0.3 Pa·s (**V6**) and the gel point from 6.8 (**V4**) to 1.1·10^1^ s (**V6**). ZnAc is used as the transesterification catalyst, which did not participate in photocuring, and can create spatial hindrances that slow photopolymerization.^31^

### 3.2 Characterization of Polymer Structure

The cross-linked structure of the polymers obtained was verified by using Soxhlet extraction (Table 3). Pure DPEPA polymer **V1** had the highest value of the gel fraction. This value gradually decreased with an increase in HPPA amount in the composition. The reason for this was the ability of HPPA, which has a single acrylic group, to form linear polymer chains in addition to being incorporated into a cross-linked polymer structure. Furthermore, the presence of extractables provided plasticization, which resulted in high flexibility of the polymers. Polymer **V6** had a lower gel fraction value than polymer **V4** due to the presence of ZnAc in the composition, which slowed photocuring, and a higher amount of branched or linear macromolecules were formed. The swelling test showed that the polymer specimens immediately started to swell after being placed in a solvent (Figure 3). The homopolymer of DPEPA **V1** had the lowest swelling value (α) in both solvents, and α increased with an increasing amount of HPPA in the composition, indicating that longer chains were formed between cross-linking points in the structure of polymer **V5**. All specimens showed the highest α values in acetone and lower α values in toluene. These results show that polymers **V1**–**V6** have a higher affinity for the polar solvent acetone than for the nonpolar solvent toluene, which makes it easier for acetone molecules to enter the polymer structure.

**Table 3:** Gel Fraction and α of Polymers

polymer	gel fraction, %	α in acetone, %	α in toluene, %
**V1**	9.4·10^1^ ± 0.1	0.6 ± 0.0	0.1 ± 0.0
**V2**	8.9·10^1^ ± 0.0	1.0·10^1^ ± 0.0	0.9 ± 0.0
**V3**	8.8·10^1^ ± 0.0	3.5·10^1^ ± 0.1	1.8 ± 0.1
**V4**	8.8·10^1^ ± 0.1	5.7·10^1^ ± 0.2	6.3 ± 0.1
**V5**	8.7·10^1^ ± 0.0	6.1·10^1^ ± 0.1	7.0 ± 0.2
**V6**	8.5·10^1^ ± 0.0	6.4·10^1^ ± 0.2	7.2 ± 0.2

**Figure 3:**
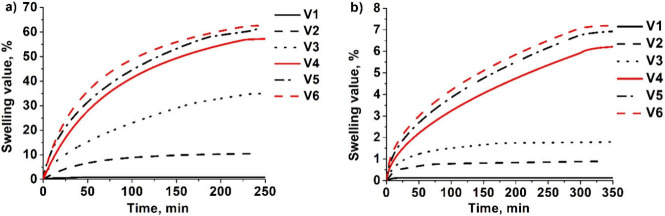
Dependence of α on the swelling time of polymers **VS1 – VS6** in polar solvent acetone (a) and in nonpolar solvent toluene (b).

### 3.3 Thermal Properties of Polymers

The thermal properties of the polymers were investigated by TGA and DMTA (Table 4, Figure 4). Polymer **V1** had the highest glass transition temperature (*T*_g_) and was more thermally stable, probably due to the higher cross-linking density confirmed by the swelling test and the higher amount of cross-linked polymer structure formed confirmed by the gel fraction. The glass transition temperature of obtained vitrimers was similar or even higher than glycerol acrylate-based vitrimer (*T*_g_ = 3.7·10^1^ °C), suitable for high resolution additive manufacturing.^32^ Polymer **V6**, prepared with ZnAc, had the lowest temperature of 10% weight loss (2.6·10^2^ °C), as well as one of the lowest glass transition temperatures (2.1·10^1^ °C), probably due to the higher amount of linear or branched macromolecule fractions.

**Table 4:** Thermal Characteristics of Polymers **V1**–**V6**

polymer	*T*_dec‑10%_, °C	*T*_g_, °C
**V1**	4.3·10^2^	5.7·10^1^
**V2**	2.9·10^2^	3.3·10^1^
**V3**	2.9·10^2^	2.6·10^1^
**V4**	2.9·10^2^	2.4·10^1^
**V5**	2.7·10^2^	2.0·10^1^
**V6**	2.6·10^2^	2.1·10^1^

**Figure 4:**
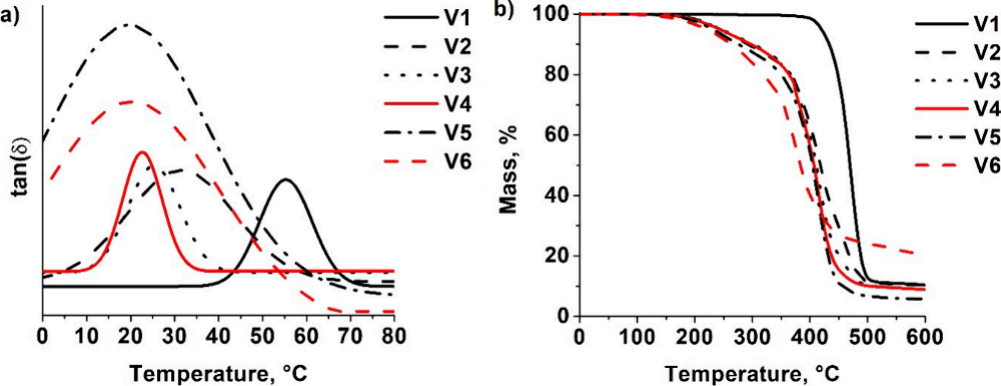
DMTA curves (a) and TGA curves (b) of polymers **V1**–**V6**.

### 3.4 Mechanical Characteristics of Polymers

The tensile test was selected to evaluate Young’s modulus (*E*), the elongation at break (*ε*) and the tensile strength (δ) of the polymers. The results are summarized in Table 5. Polymer **V1** had the highest value of *E* and δ and the lowest *ε* value. This indicates that polymer **V1** is a stiff and rigid material at room temperature. These results correlate with the high value of the gel fraction, which confirms the formation of short chains between cross-linking points in the polymer **V1** structure. The homopolymer of HPPA **V5** had the lowest value of *E* and δ, and the highest value of *ε*, indicating that it is a soft and flexible material. Furthermore, the addition of HPPA in the composition increased the flexibility of the polymers and decreased their tensile strength. For example, when the amount of HPPA increased from 0 mol (**V1**) to 90 mol (**V4**) δ was reduced from 8.8 to 0.6 MPa and *ε* increased from 0.3% to 72.2%. ZnAc had almost no influence on the *E*, δ and *ε* of polymers, because when comparing the polymers **V4** and **V6**, only a small difference in their values was observed. In addition, mechanical characteristics of obtained polymers were similar or higher than biobased vitrimer with glycerol acrylate fragments (*E* = 1.3 MPa) suitable for digital light processing 3D printing.^26^

**Table 5:** Mechanical Characteristics of Polymers

polymer	*E*, MPa	δ, MPa	ε, %
**V1**	9.2·10^2^ ± 0.3	8.8 ± 0.3	0.3 ± 0.0
**V2**	2.1·10^2^ ± 0.0	2.2 ± 0.1	2.6 ± 0.0
**V3**	1.2·10^1^ ± 0.0	1.9 ± 0.0	4.4·10^1^ ± 0.1
**V4**	2.0 ± 0.1	0.6 ± 0.0	7.2·10^1^ ± 0.2
**V5**	0.6 ± 0.0	0.2 ± 0.0	1.1·10^2^ ± 0.0
**V6**	1.9 ± 0.1	0.3 ± 0.0	7.5·10^1^ ± 0.2

### 3.5 Relaxation of the Stress of the Vitrimers

Vitrimers have dynamic bonds that can rearrange the network and reduce the relaxation of internal stress caused by deformation. Topology rearrangements were investigated by measuring the stress relaxation of polymers **V4** and **V6**, and the topology freezing transition temperature (*T*_v_) was determined from the Arrhenius curves for relaxation times of 10^6^ s (Figure 5).^16^ Zinc acetylacetone hydrate was used as a transesterification catalyst in composition **V6**, while composition **V4** was left without a catalyst. As a result, the topology freezing transition temperature of vitrimer **V6** (*T*_v_ = 1.1·10^2^ °C) was lower and the relaxation times were shorter compared to those of vitrimer **V4** (*T*_v_ = 1.4·10^2^ °C).

**Figure 5:**
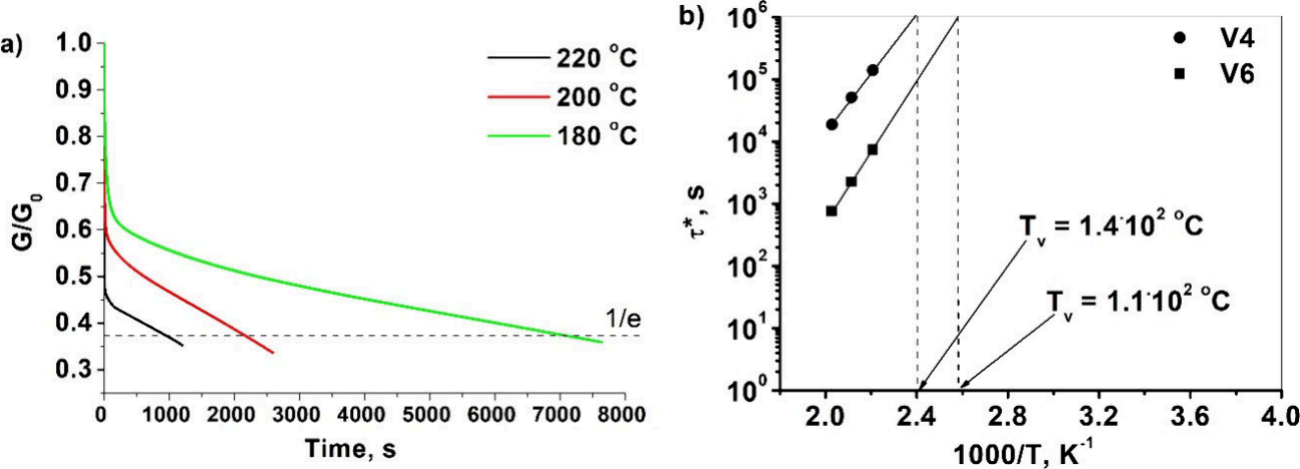
**V6** stress relaxation curves vs time (a) and the Arrhenius plot of relaxation times of **V4** and **V6** (b).

### 3.6 Shape-Memory Properties

Both vitrimers **V4** and **V6** showed thermoresponsive shape-memory properties, which were determined by their *T*_g_ and *T*_v_. Unlike other polymers, vitrimers can maintain two permanent shapes. The shape-memory behavior of vitrimer **V4** is presented in Figure 6. To obtain the first temporary shape, the specimen must be heated to a temperature above its *T*_v_ and reshaped and cooled to a temperature lower than T_v_ to fix its temporary shape. To obtain the second temporary shape, the specimen has to be heated to a temperature higher than that of *T*_g_, reshaped, and cooled to a temperature lower than that of *T*_g_. Both vitrimers **V4** and **V6** could easily recover their permanent shapes when necessary and maintain their temporary shapes.

**Figure 6:**
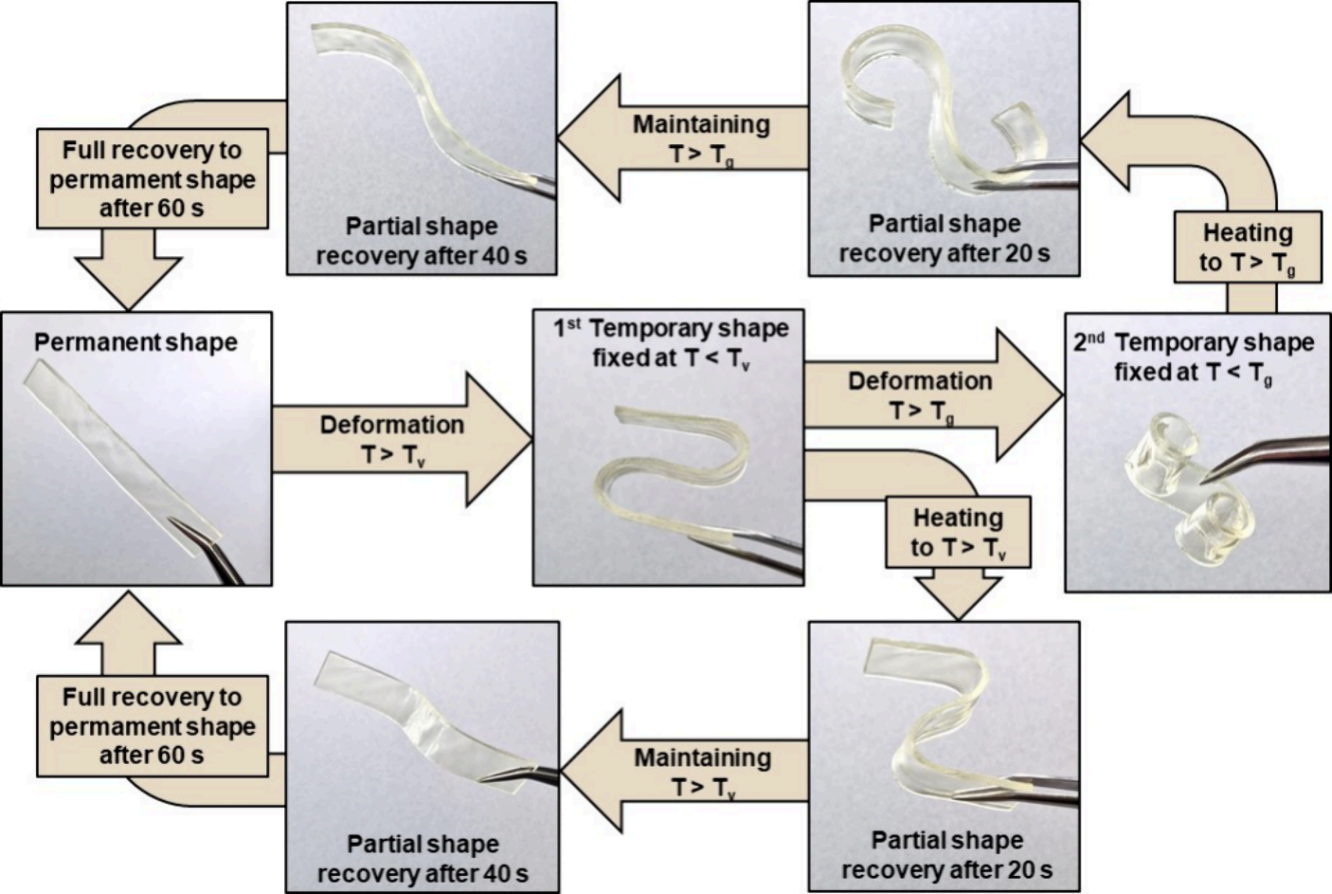
Shape-memory behavior of the **V4** vitrimer sample, representing temporary shape fixity and permanent shape recovery

### 3.7 Self-Welding properties

For the investigation of self-welding properties, the tensile test was carried out to compare the *E*, δ and ε of vitrimers before and after the self-welding test. Furthermore, to estimate the effect of temperature on self-welding properties, two different temperatures were chosen: the first was 10 ± 5 °C higher than their *T*_v_, and the second was 40 ± 5 °C higher than their vitrimer *T*_v_. The images of the vitrimer **V4** and **V6** samples, the stress–strain curves and mechanical characteristics are presented in Figure 7. The *E* of vitrimer **V4** was increased slightly from 2.0 to 2.5 MPa and δ increased from 0.6 to 1.1 MPa while *ε* was reduced from 7.2·10^1^ to 7.1·10^1^% after self-welding at 150 °C (Figure 7c). Similar results were obtained after self-welding at 180 °C, the value of *E* and δ remained almost the same (2.5 and 1.2 MPa, respectively), while *ε* slightly increased to 7.1·10^1^%, indicating that self-welding did not significantly worsen its mechanical properties. Similarly to vitrimer **V4**, *E* of vitrimer **V6** was increased slightly from 1.9 to 2.3 MPa and δ increased from 0.3 to 0.9 MPa, while *ε* was reduced from 7.5·10^1^ to 7.3·10^1^% after self-welding at 125 °C. Different results were observed after self-welding of vitrimer **V6** at 150 °C. *E* increased from 1.9 to 49.4 MPa and δ increased from 0.3 to 1.5 MPa, while *ε* was reduced from 7.5·10^1^ to 2.6·10^1^%. These changes were due to topological rearrangements and the possible thermal polymerization of unreacted functional groups. Additionally, the presence of the catalyst in vitrimer **V6** assisted reactions at high temperature and consequently affected remarkably mechanical characteristics. Similar increase in *E* and δ values after self-welding were reported in the litherature for glycerol acrylate-based vitrimer and *E* value was increased by 6 times, while δ value increased 2 times compared to original sample.^26^ Vitrimer **V6** had a higher value of *E* than vitrimer **V4** after self-welding (at 150 and 180 °C, respectively) due to different T_v_ values and the presence of the catalyst. Stress relaxation times of vitrimers depend on the temperature and shorten when the temperature is increased, as visible in stress relaxation curves. Because of that, transesterification reactions occurred faster in vitrimer **V6** sample and a higher value of Young’s modulus of vitrimer **V6** was observed. However, it is important to note that vitrimer **V4** was able to demonstrate excellent self-welding properties, in a short period of time (30 min) at relatively low temperature (150 °C) with almost no change in mechanical properties, which is an advantage since self-welding is used more often for repairing products rather than producing new ones. Such vitrimers are promising materials because the use of catalysts raises health and environmental concerns, as they are usually toxic substances, and therefore the scientific community is looking for catalyst-free systems.^13^

**Figure 7:**
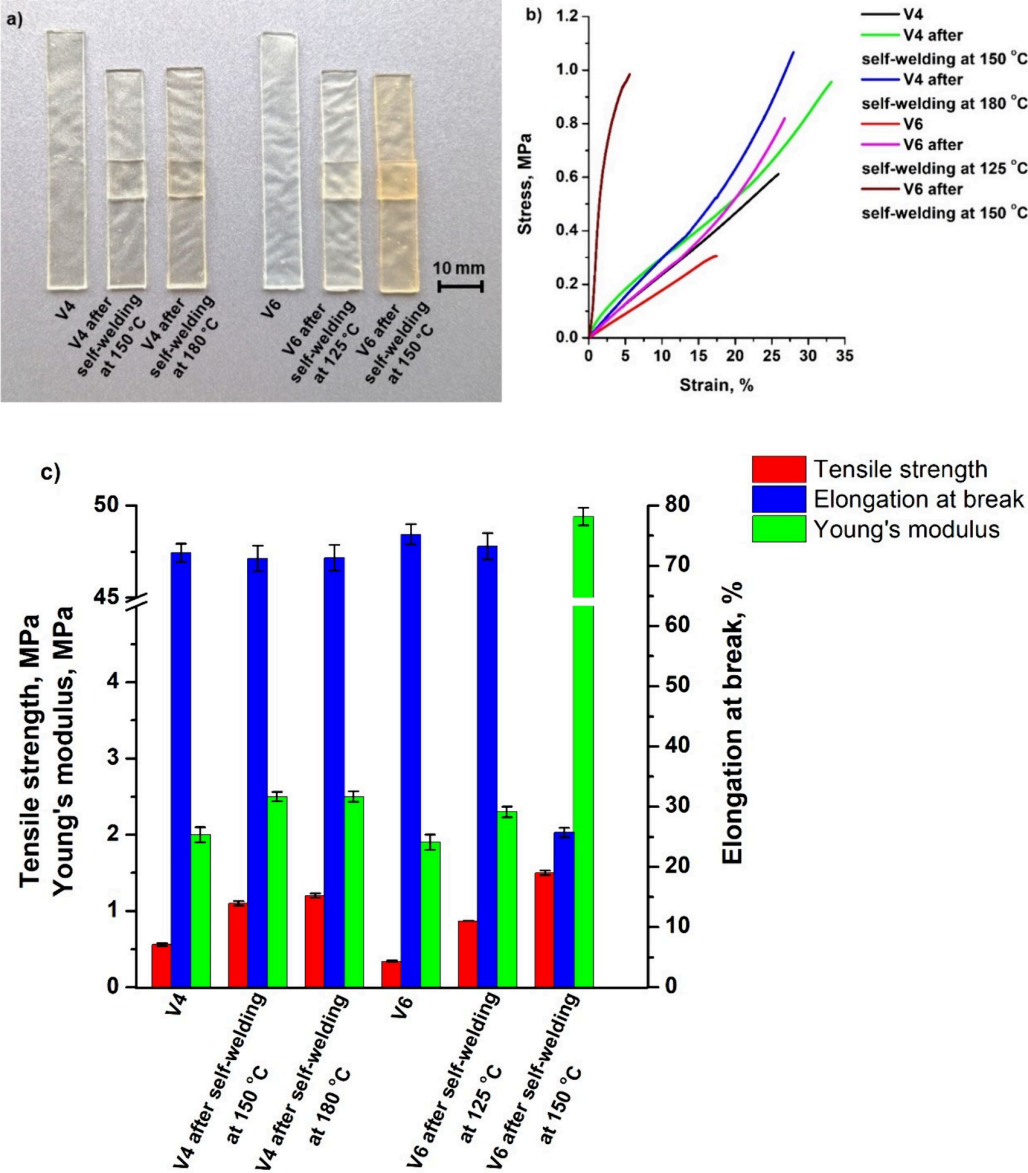
Vitrimers **V4** and **V6** sample images (a), their tensile stress–strain curves (b), and their mechanical characteristics obtained from a tensile test (c) before and after self-welding tests.

### 3.8 Thermal Reprocessing

Reprocessability allows for the production of new polymer products using product waste. Three cycles of hot-press reprocessing were performed, and numbers 1, 2, or 3 were added to the vitrimer codes indicating the first (**V4.1** and **V6.1**), second (**V4.2** and **V6.2**), and third (**V4.3** and **V6.3**) cycle. To evaluate the reprocessability of vitrimers, a tensile test was performed to compare *E*, δ and *ε* of vitrimer samples before and after hot-press reprocessing. The FT-IR spectra of vitrimers before and after each cycle of reprocessing are presented in Supplementary Figure S2. No changes in the spectra were observed after the three cycles of reprocessing for both vitrimers, indicating that no structural changes occurred. Also, reprocessing had no significant effect on the yield of insoluble fraction, but increased the cross-linking density of the vitrimers (Table S1). Images, stress–strain curves, and mechanical characteristics of the vitrimers **V4** and **V6** samples are presented in Figure 8. The *E* of vitrimer **V4** was increased from 2.0 to 10.5 MPa and δ was increased from 0.6 to 2.7 MPa, while ε was reduced from 7.2·10^1^ to 5.4·10^1^% (Figure 8 c) after the first cycle of hot-press reprocessing. After further cycles, *E* of vitrimer **V4.1** gradually increased from 10.5 to 12.2 (**V4.2**) and 46.9 MPa (**V4.3**) while ε reduced from 5.4·10^1^ to 2.3·10^1^ (**V4.2**) and 1.8·10^1^% (**V4.3**), indicating that the reprocessability increased the mechanical strength of vitrimer **V4**. The *E* of vitrimer **V6** increased from 1.9 to 5.9 MPa and δ was increased from 0.3 to 1.1 MPa while ε was reduced from 7.5·10^1^ to 5.7·10^1^% after the first cycle of hot-press reprocessing. After further cycles, the *E* of vitrimer **V6.1** gradually increased from 5.9 to 7.7 (**V6.2**) and 1.5·10^1^ MPa (**V4.3**) while ε reduced from 5.7·10^1^ to 4.3·10^1^ (**V6.2**) and 2.9·10^1^% (**V6.3**). Similar to HPPA-based vitrimers, a gradual increase in *E* and δ values, as well as a gradual decrease in ε values, has been previously reported in biobased vitrimers with glycerol moieties after the first, second, and third recycling cycles.^33^ These changes in the mechanical properties of both vitrimers were due to topological rearrangements during processing. The values of *E* and δ of vitrimer **V4** were almost 3 times higher than those of vitrimer **V6** after the first cycle of hot-press reprocessing. The reason for this was the different temperature regime during the reprocessing test than during the self-welding test. During the reprocessing test, different temperatures were used for different vitrimers, which were 70 ± 5 °C higher than their T_v_. The heating time was the same for both vitrimers. The results show that the transesterification reactions proceeded more efficiently in vitrimer **V4** during reprocessing compared to the self-welding test and, therefore, higher values of *E* and δ were obtained. In addition, the lower temperature was chosen for vitrimer **V6** due to its lower thermal stability determined by the TGA test. A higher reprocessability temperature could result in thermal degradation of polymer **V6** as it had a lower temperature of 10% weight loss than vitrimer **V4** (2.6·10^2^ and 2.9·10^2^ °C, respectively). These results show that thermal reprocessing is an excellent method for producing new products with improved mechanical strength from vitrimer **V4** and **V6** waste.

**Figure 8:**
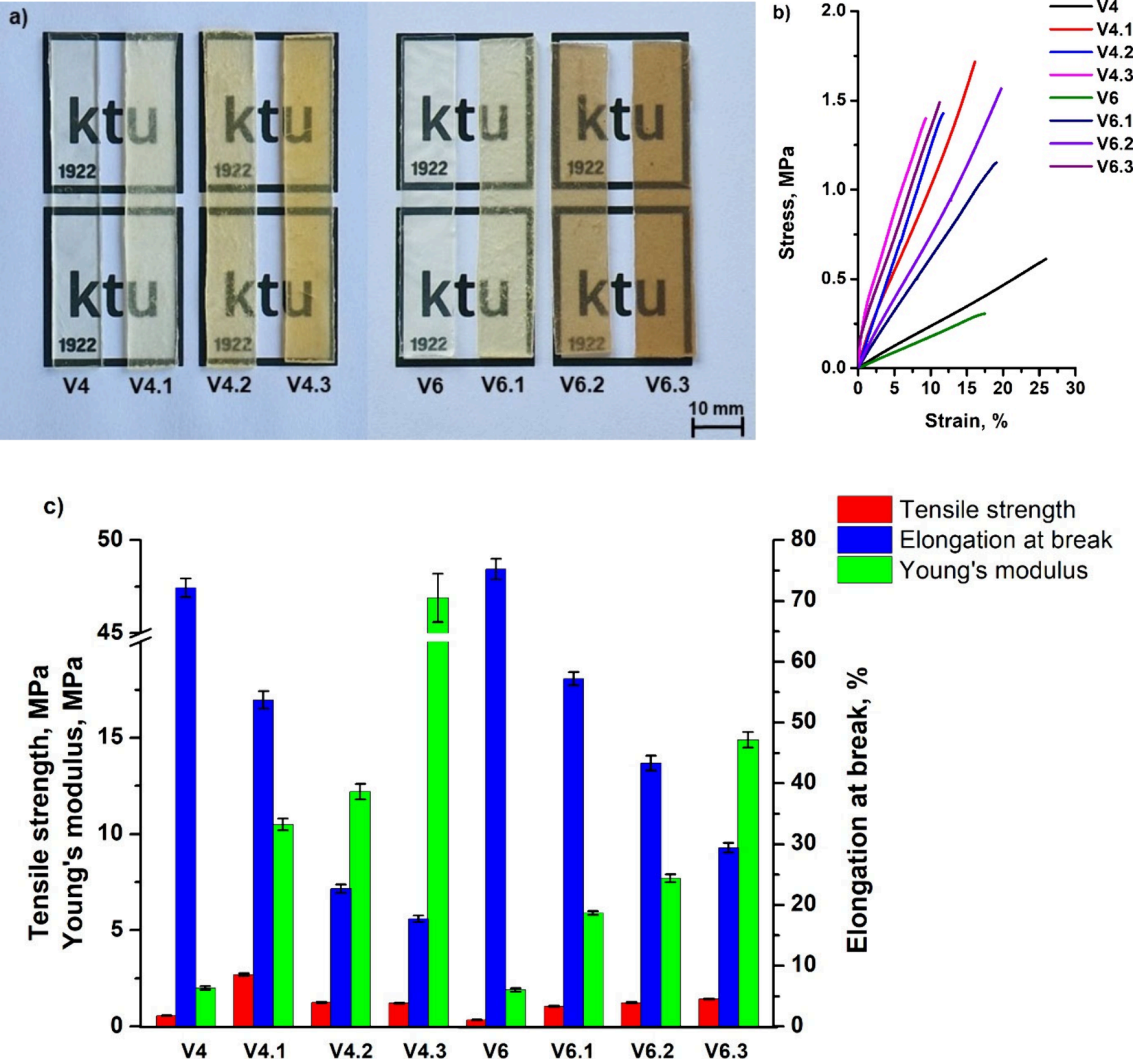
Vitrimers **V4** and **V6** sample images (a), their tensile stress–strain curves (b), and their mechanical characteristics obtained from a tensile test (c) before and after hot-press reprocessing.

### 3.9 Antimicrobial Properties

The antibacterial and antifungal activity of the polymers was investigated after 1 h contact time of the polymer samples with microbial suspensions to determine their dependence of activity on the polymer structure. This short period of time compared to standard 24 h time and almost 10 times higher concentration of CFU/mL (ISO 16869:2008 ^34^ and ISO 22196:2011 ^35^) was chosen to prove that polymers **V1**-**V6** are highly antimicrobial materials and can greatly reduce the viability of bacteria and microscopic fungi.

Two Gram-positive bacteria, *B. subtilis* and *S. aureus*, and two Gram-negative bacteria, *P. aeruginosa* and *E. coli*, were selected for the investigation of the antibacterial properties of polymers. These bacteria were chosen because they can cause various infections and can be transmitted through human contact or from infected surfaces.^36^ *P. aeruginosa*, *E. coli*, and *S. aureus* are common antibiotic resistant bacteria that cause most hospitally aquired infections and can cause serious health problems such as sepsis, mastitis, bloodstream infections, or pneumonia.^37,38^ *B. subtilis* is considered nonpathogenic bacteria, although it can cause secondary healthcare-associated infections that can be lethal to immunocompromised patients.^39^

The test results are presented in Figure 9. In all cases, antibacterial activity of polymers **V1**-**V6** increased with an increasing amount of HPPA fragments in the polymer structure. For example, when the amount of HPPA was increased from 10 mol (**V2**) to 30 mol (**V3**), the reduction in *S. aureus* cells was increased from 3.1·10^1^% to 4.7·10^1^%. The reason for this could be the aromatic structure of HPPA, as it is known that aromatic groups have antibacterial properties.^40^ The highest antibacterial activity was observed against Gram-positive bacteria *B. subtilis* and was in the range of 9.9·10^1^ to 1.0·10^2^% and the lowest antibacterial activity was observed against Gram-positive *S. aureus* and was in the range of 2.9·10^1^ to 5.2·10^1^%. The reason may be due to the morphological differences: *S. aureus* is rounded and can form clusters, whereas *B. subtilis* is rod-shaped and flagellated. Among Gram-negative bacteria, the reduction in *P. aeruginosa* cells was lower compared to that in *E. coli*. For example, polymer **V5** reduced the viability of *E. coli* cells by 7.5·10^1^% while the viability of *P. aeruginosa* was only reduced by 5.4·10^1^%. The reason may be the ability of *P. aeruginosa* to aggregate into enduring biofilms.^41^ ZnAc also affected the antibacterial activity of the materials. Antibacterial activity increased from 7.4·10^1^% to 9.5·10^1^% against *E. coli*, and from 4.7·10^1^% to 8.4·10^1^% against *P. aeruginosa* by adding 5 wt% of ZnAc to the composition due to the high antibacterial activity of zinc.^42^

**Figure 9:**
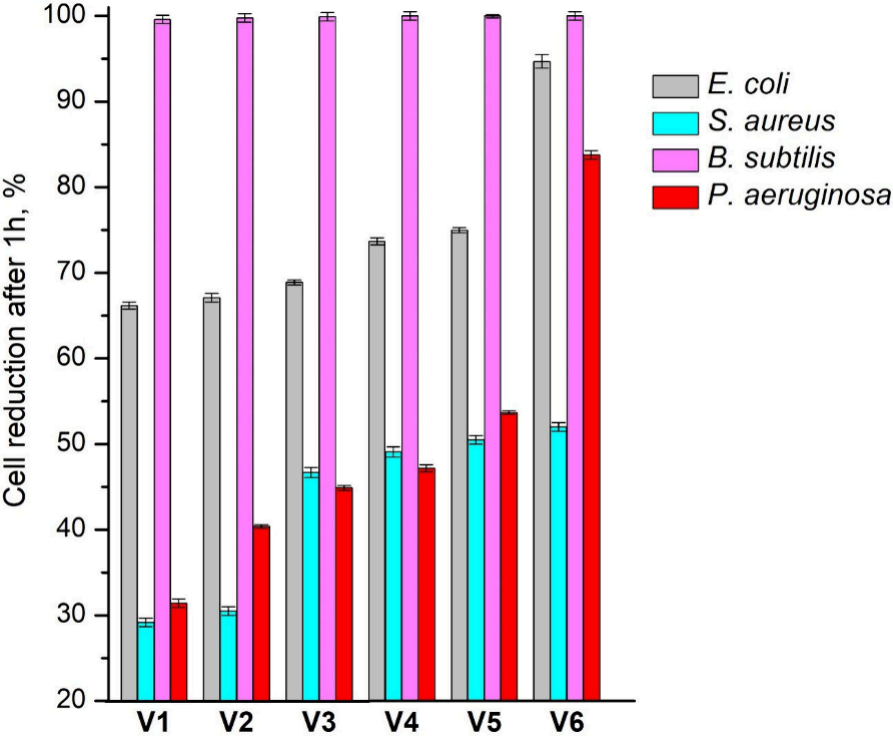
Antibacterial activity of polymers against *B. subtilis*, *S. aureus*, *P. aeruginosa*, and *E. coli* after 1 h of contact.

The antifungal activity of polymers was tested against four microscopic fungi, *A. flavus*, *A. niger*, *C. cladosporioides*, and *S. brevicaulis*. *A. flavus* is commonly found in the soil and was selected because it can cause direct infections and aspergillosis in patients with a weakened immune system.^43^ *A. niger* is a common food spoilage microorganism found on the surfaces of various fruits and vegetables and can also cause pneumonia.^44^ *C. cladosporioides* are commonly found in decomposing organic materials or as food contaminants. They are human pathogens that cause eye or skin infections.^45^ *S. brevicaulis* is an emerging pathogen that causes mycosis mainly in soft tissues and the lungs. The number of infections caused by *S. brevicaulis* is reported to have increased in recent decades.^46^

The test results are presented in Figure 10. In all cases, antifungal activity increased with the increase in the amount of HPPA fragments in the polymer structure due to the antifungal aromatic group present in the HPPA structure.^47^ *Aspergillus* is more resistant to antifungal materials compared to the other fungal genus.^48^ Because of that, cell reduction after 1 h was in the range of 4.6·10^1^-6.1·10^1^% against *A. flavus* and 6.4·10^1^-8.0·10^1^% against *A. niger* and was lower compared to other microscopic fungi. The highest antifungal activity was observed against *C. cladosporioides* (9.2·10^1^ to 9.4·10^1^%) and *S. brevicaulis* (9.0·10^1^ to 9.3·10^1^%) with a difference of less than 4%. Furthermore, vitrimer **V6** with 5 wt% of ZnAc showed higher antifungal activity than polymer **V4** without ZnAc due to the antifungal activity of zinc.^49^

**Figure 10:**
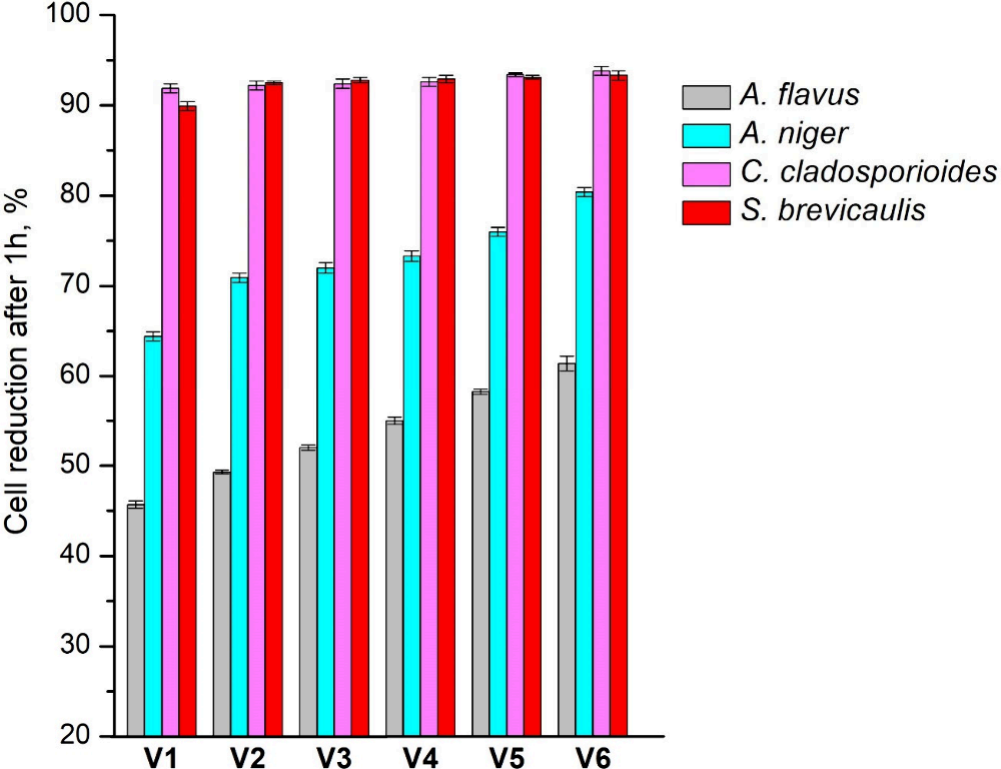
Antifungal activity of polymers against *A. flavus*, *A. niger*, *C. cladosporioides*, and *S. brevicaulis* after 1 h of contact

In summary, all polymers, **V1**–**V6**, showed antibacterial and antifungal activity, even after 1 h of contact time. It is important to note that polymers are permanently antimicrobial through all of their volume. The antimicrobial activity remains the same even if the surface of the product is scratched or damaged in other ways. This is a huge advantage over other antimicrobial materials, which usually provide only temporary antimicrobial activity. For example, disinfectant solutions must be applied periodically as their effect is instantaneous but not persistent, essential oils evaporate and inorganic particles rub off over time, gradually reducing the antimicrobial effect.^50,51^ Because of this, polymers with antimicrobial structural fragments are the easiest solution for permanent antimicrobial activity with the lowest maintenance costs, which is very important for areas with high microbe concentrations, such as medical facilities.^52^

### 3.10 LCD 3D Printing

The printability of resin **V2** was tested by using a Zortrax Inkspire LCD 3D printer, and a Y-shaped connector (Figure 11) was successfully printed, showing that the selected resin is suitable for this technology. Optical 3D printing is a popular technology in medical, aerospace, automotive, electronics, architecture, and other fields.^53^ It enables the printing of custom products with small parts and a smooth surface finish, which is impossible to obtain with other 3D printing techniques.^54^ 3D printing of medical tools allows for personalized and precise treatments, since custom tools for individual patients or procedures can be easily printed.^55^ It is also used to print custom organs of patients that are used practicing before complicated surgeries, or to print custom prosthetic limbs, or to create of more realistic educational medical devices.^56^ Resin **V2** provides the opportunity to choose a more environmentally friendly resin for optical 3D printing, as most commercially available resins are petroleum-based and are toxic to humans and hazardous to aquatic organisms with long-lasting effects.^57^

**Figure 11:**
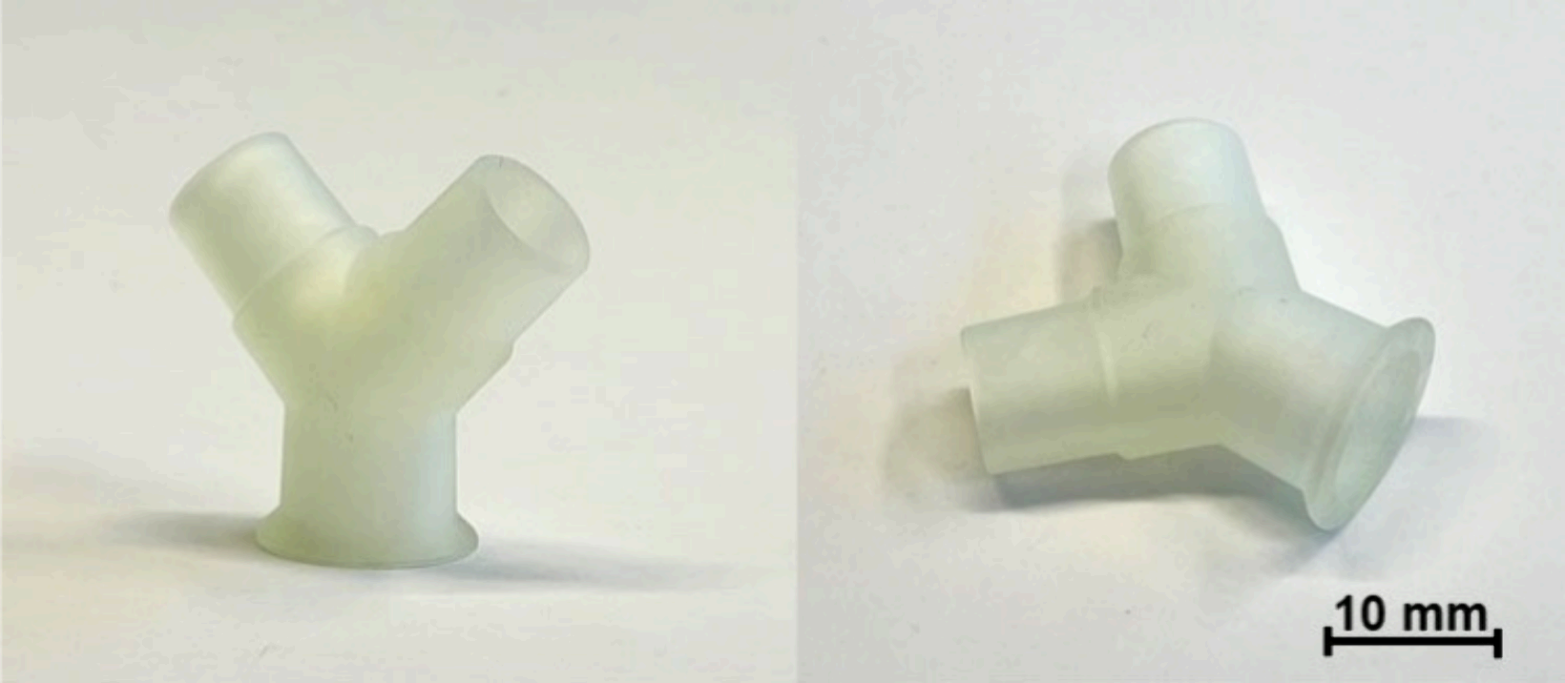
LCD 3D printed Y-shaped connector from **V2** resin.

## Conclusions

In this study, new vitrimers composed of dipentaerythritol pentaacrylate and 2-hydroxy-3-phenoxypropyl acrylate were synthesized, and their properties were investigated. Main conclusions are summarized as follows:

- The results of the real-time photorheometry showed that when the amount of 2-hydroxy-3-phenoxypropyl acrylate increased from 10 to 90 mol, the shrinkage and viscosity of the resin decreased from 1.8·10^1^ to 1.2·10^1^% and 0.3 to 0.2 Pa·s respectively, but the rigidity was reduced (Ǵ reduced from 2.4·10^1^ to 1.4·10^1^ MPa) and the gel point increased from 2.0 to 6.8 s, indicating that 2-hydroxy-3-phenoxypropyl slows the photocuring process.
- When the amount of dipentaerythritol pentaacrylate was increased from 0 to 1 mol, the glass transition temperature of the polymers increased from 2.0·10^1^ to 3.3·10^1^ °C, and the temperature of the 10% weight loss increased from 2.7·10^2^ to 2.9·10^2^ °C. Young’s modulus values also increased from 0.6 to 213.3 MPa, while elongation at break decreased from 1.1·10^2^ to 2.6%.
- All vitrimers exhibited thermoresponsive shape memory properties, maintaining two temporary shapes and a permanent shape.
- Vitrimers showed self-welding properties. Vitrimer prepared without catalyst had very similar mechanical characteristics before and after self-welding, indicating that self-welding had no impact on its properties. However, the value of Young’s modulus increased 26 times after self-welding for vitrimer prepared with catalyst, indicating that catalyst assisted reactions at high temperatures.
- Vitrimers showed impressive reprocessability properties. Young’s modulus and tensile strength of the catalyst-free vitrimer increased 5 times after hot-press reprocessing, while Young’s modulus and tensile strength of the catalyst-containing vitrimer increased only 3 times, indicating that thermal reprocessability increases the values of Young’s modulus and tensile strength of polymers.
- All polymers showed antimicrobial activity even after a short period of time (1 h) against not only standard microbial strains (*E. coli*, *S. aureus*, *A. flavus*, and *A. niger*), but also against other widely spread microbial strains (*B. subtilis*, *P. aeruginosa*, *C. cladosporioides*, and *S. brevicaulis*). The antibacterial and antifungal activity of the polymers increased with increasing amount of 2-hydroxy-3-phenoxypropyl acrylate in the composition, as well as due to the presence of a transesterification catalyst containing Zn.
- The resin composed of 1 mol of dipentaerythritol pentaacrylate and 10 mol of 2-hydroxy-3-phenoxypropylacrylate was applied to LCD 3D printing technology using the Zortrax Inkspire printer equipped with a 405 nm light source. LCD 3D printed Y-shaped connector showed high accuracy and an even surface finish, showing that selected resin is suitable for this technology.

The results obtained offer valuable insights into the development of photocured vitrimers and catalyst-free vitrimers with excellent self-welding and reprocessability properties. The suitability of the resin for LCD 3D printing expands its areas of application in the fields of medicine, aerospace, automotive, electronics, architecture, and others. Additionally, antimicrobial activity makes these vitrimers especially promising for application in areas with high concentrations of microbes such as medical facilities.

## Supplementary Material



## Notes

Detailed characterization techniques; Experimental methodologies; FT-IR spectra (PDF)
