# Supplementary material for: Antimicrobial Vitrimers Synthesized from Dipentaerythritol Pentaacrylate and 2‑Hydroxy-3-phenoxypropyl Acrylate for LCD 3D Printing
Source: Biomacromolecules. 2025 Jun 24;26(7):4584–94. doi: 10.1021/acs.biomac.5c00577 (PMC12264950; doi:10.1021/acs.biomac.5c00577)
Supplement: Supplementary file 1 [file bm5c00577_si_001.pdf]

Antimicrobial Vitrimers Synthesized from Dipentaerythritol Pentaacrylate and 2-Hydroxy-3-Phenoxypropyl Acrylate for LCD 3D Printing

Vilte Sereikaite[1], Aukse Navaruckiene[1], Vita Raudoniene[2], Danguole Bridziuviene[2], Paulius Cerkauskas[3], Saulius Lileikis[3], Kastytis Pamakstys[4], Egidija Rainosalo[5], Anne-Sophie Schuller[6], Christelle Delaite[6], Jolita Ostrauskaite[1,]*

[1] Department of Polymer Chemistry and Technology, Kaunas University of Technology, Radvilenu Rd. 19, LT-50254 Kaunas, Lithuania

[2] Biodeterioration Research Laboratory, State Scientific Research Institute Nature Research Center, Akademijos St. 2, LT-08412 Vilnius, Lithuania

[3] JSC 3D Creative, Mokslininku St. 2, LT-08412 Vilnius, Lithuania

[4] Institute of Environmental Engineering, Kaunas University of Technology, Gedimino St. 50, LT-44239, Kaunas, Lithuania

[5] Centria University of Applied Sciences, Talonpojankatu 2, FI-67100 Kokkola, Finland

[6] Laboratoire de Photochimie et d'Ingénierie Macromoléculaires - EA4567, Université de Haute Alsace, Université de Strasbourg, 3b rue Alfred Werner, 68093 Mulhouse

Cedex, France

*Correspondence: jolita.ostrauskaite@ktu.lt

S.1. Characterization Techniques

The UV/Vis cure tests of the resins were performed on an MCR302 rheometer (Anton Paar) equipped with the plate/plate measuring system. The measurement gap was set to 0.1 mm and the samples were irradiated with UV/Vis light in a wavelength range of 250–450 nm through the glass plate using the OmniCure S2000 UV/Vis spot curing system (Lumen Dynamics Group Inc.). The shear mode was used with a frequency of 10 Hz and a shear strain of 1%.

Fourier transform infrared spectroscopy (FT-IR) spectra were recorded using a Perkin Elmer Spectrum One FT-IR spectrometer (Perkin Elmer). The spectra obtained have been normalized. The range of wavenumbers was (650-4000) cm^{-1}.

The Soxhlet extraction was used to determine the yield of insoluble fraction. Polymer samples of 0.4 g were extracted with acetone for 24 h, dried under vacuum until no changes of the weight were observed and the yield of insoluble fraction was calculated.

Cross-linking density was calculated based on the theory of rubber elasticity, using the following equation:

$$\sigma = NRT(\frac{\lambda}{\lambda^2}),$$

where σ is stress (MPa), N is cross-linking density (mol/m^3), R is the universal gas constant (8.314 J/Kmol), T is temperature (K), λ is strain (the same $\lambda = 1.2\%$ was taken for the calculation of the cross-linking density for all synthesized polymers).[1]

The swelling values of cross-linked polymer samples were obtained by measuring the mass of the samples swollen in acetone and toluene every 10 minutes at 25 °C until no mass change was

observed. The swelling value was calculated as a percentage change in mass. Polymer specimens of 0.5 g were used for the swelling test. The swelling value was calculated using the following equation:

$$\alpha = \frac{m - m_0}{m_0} \cdot 100,$$

where α is the swelling value (%); m is the mass of swollen specimen (g); and m_0 is the initial mass of specimen (g).

Thermogravimetric analysis (TGA) was performed on a TGA Q500 apparatus (TA Instruments) with a heating rate of 20 °C/min under nitrogen atmosphere (20 mL/min). The temperature range of 20−800 °C was used.

Dynamic mechanical thermal analysis (DMTA) was performed on an MCR302 rheometer (Anton Paar). The temperature was increased from 0 °C to 80 °C with a heating rate of 2.0 °C/min. The normal force was set at 5 N during the measurement, shear mode with a frequency of 1 Hz and a shear strain of 0.1% were used.

Mechanical properties of the synthesized polymers were determined by the tensile test performed on a Testometric M500-50CT testing machine (Testometric Co Ltd.) at room temperature (25°C). The dimensions of the test specimens were 70 (±0.00) x 10 (±0.00) x 1 (±0.10) mm. The test was performed at the tensile speed of 5 mm/min until the break of the specimen. Five parallel specimens were used for this test.

The topology freezing temperature (T_v) was determined by stress relaxation experiments on a MCR302 rheometer. The samples were equilibrated with the selected measurement temperature (180–220 °C) for 10 min and decreasing stress was recorded over time.

The self-welding experiment was carried out by cutting the UV-cured sample into two equal pieces which were placed on top of each other with 1 cm^2 overlap and heated in the temperature

chamber at 125, 150 and 180 °C for 30 min. No additional pressure was applied to the samples. After that, samples were cooled down to room temperature, and a tensile test was performed.

Vitrimer samples were frozen in the refrigerator and crushed into fine powder using mortar and pestle. The vitrimer powder was kept in a heating chamber at 60 °C for 4 hours to remove moisture. The Lap 40 press (Gottfried Joos Mschinenfabrik GmbH & Co. kg) was used for the reprocessability test. 7 g of powder were placed in a (70 x 70 x 1) mm stainless steel frame, and heated at a temperature above T_v for 30 min (**V4** at 170 °C and **V6** at 140 °C), then temperature was increased to 210 °C for **V4** and to 180 °C for **V6**, and heating was continued for another 30 min. The applied pressure was 2 MPa. After that, the heating was turned off and the samples were allowed to cool to room temperature (25 °C) before being removed from the press. The samples were cut to rectangular shapes of (70 x 10 x 1) mm and a tensile test was performed.

The study of the antimicrobial activity of polymers was performed using four strains of bacterium, Gram-negative bacterium *Escherichia coli* ATCC 25922 (*E. coli*) and *Pseudomonas aeruginosa* ATCC 15442 (*P. aeruginosa*) and Gram-positive bacterium *Staphylococcus aureus* ATCC 29213 (*S. aureus*) and *Bacillus subtilis* ATCC 6633 (*B. subtilis*), and four fungal strains *Aspergillus niger* MUCL 19001 (*A. niger*), *Aspergillus flavus* CBS 120264 (*A. flavus*), *Cladosporium cladosporioides* MUCL 926 (*C. cladosporioides),* and *Scopulariopsis brevicaulis* MUCL 14213 (*S. brevicaulis*). The final inoculum concentrations were as follows: 3.5×10^6 colony forming units/mL (CFU/mL) for *E. coli*, 4.0×10^6 CFU/mL for *P. aeruginosa,* 8.0×10^6 CFU/mL for *S. aureus*, 3.5×10^6 CFU/mL for *B. subtilis,* 1.5×10^5 CFU/mL for *A. niger*, 1.4×10^5 CFU/mL for *A. flavus*, 1.0×10^5 CFU/mL for *C. cladosporioides*, and 9.5×10^5 CFU/mL for *S. brevicaulis.* The test films 10 (±0.00) x 10 (±0.00) x 0.5 (±0.15) mm were placed in empty sterile

Petri dishes and inoculated with microbial suspension (10 μl). The methodology was described previously.[2]

The Microsoft Excel program ANOVA was used for the statistical analysis of the collected data. The estimated *p*-value was below 0.05 within the groups.

S.2. LCD 3D Printing

The 3D printer Zortrax Inkspire with a 405 nm LED source was used for polymer sample printing. The printing volume was 192 x 120 x 280 mm, the layer thickness was 50 μm, layer exposure time was 12 s, the exposure time of the bottom layers was 120 s (number of bottom layers was 5), and the exposure off time was 5 s. After printing, polymer samples were washed with isopropyl alcohol and post-cured for 6 min under 405 nm UV lamp.

S.3. Preparation of Photopolymer Specimens

The initial mixtures containing 0-1 mol of DPEPA, 0-90 mol HPPA, 3 mol% of photoinitiator TPOL, and 0-5 wt.% of ZnAc were stirred with magnetic stirrer at 40 °C for 10 min. When the homogeneous mixture was obtained, the resin was poured into Teflon mold (70 x 10 x 0.5 mm) and cured for 3-5 min in the UV/Vis irradiation chamber BS-02 (Opsytec Dr. Grobel) with an intensity of 30 mW/cm^2 and a wavelength range of 280–400 nm.

S.4. Characterization of Polymer Structure

The chemical structure of the polymers **V1 – V6** was confirmed by FT-IR spectroscopy (**Figure S1**). The signals of the C=C group that are present at 1627-1632 cm^{-1} in the DPEPA and HPPA spectra were reduced in polymer spectra. This indicates that polymers were formed during photocuring. Furthermore, the signal of the unreacted C=C group, visible in the spectrum of polymer **V1,** was reduced by adding HPPA (polymer **V2**) and gradually decreased with further increase of HPPA amount (polymer **V3** – **V6**). This confirms that HPPA can overcome spatial

hindrances of DPEPA and react with DPEPA unreacted acrylic groups. The other characteristic group signals were also visible in the spectra: OH at 3426-3471 cm^{-1}, C-O-C at 1048-1057 cm^{-1}, C=O at 1718-1727 cm^{-1}, aromatic C-H at 3045-3053 cm^{-1}, aromatic δ C-H at 685-689 cm^{-1}, aliphatic C-H at 2936-2945 cm^{-1}, and vinyl C-H at 1396-1405 cm^{-1}.

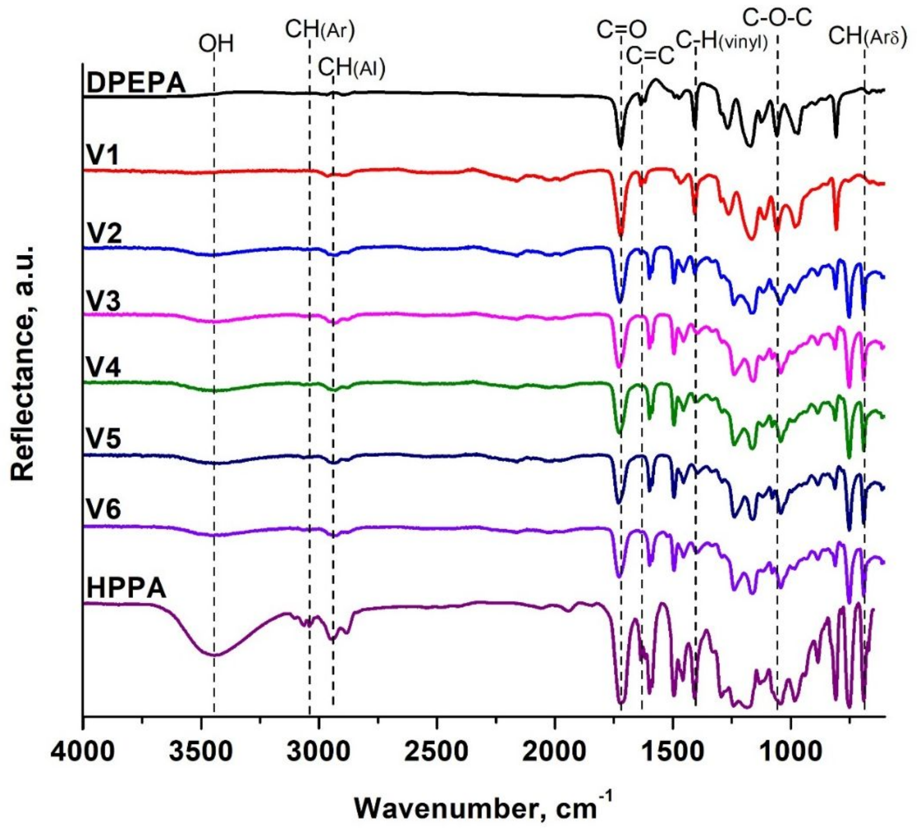

Figure S1. FT-IR spectra of DPEPA, HPPA and polymers **V1 – V6**

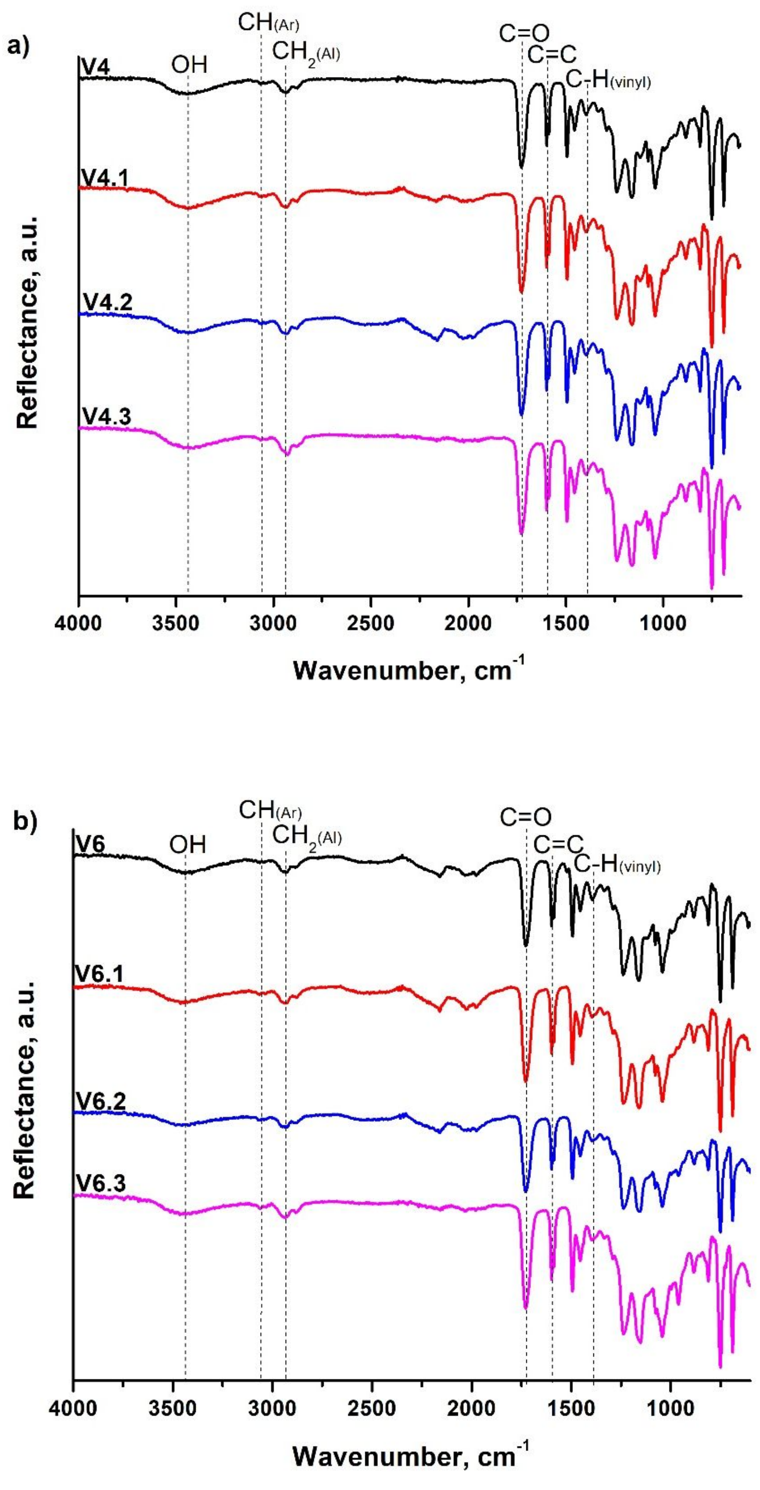

Figure S2. FT-IR spectra of vitrimers **V4** (a) and **V6** (b) before and after hot press reprocessing

Table S1. Gel fraction and cross-linking density of polymers before and after reprocessing.

Polymer	Gel fraction, %	Cross-linking density, mol/m^3
**V4**	87.5 ± 0.5	26.1 ± 1.3
**V4.1**	84.0 ± 0.3	121.1 ± 6.1
**V4.2**	84.2 ± 0.2	131.8 ± 6.3
**V4.3**	84.1 ± 0.4	287.4 ± 9.2
**V6**	85.0 ± 0.0	17.8 ± 0.9
**V6.1**	81.3 ± 0.2	66.1 ± 2.8
**V6.2**	81.0 ± 0.1	86.9 ± 3.4
**V6.3**	81.1 ± 0.3	226.9 ± 8.4

REFERENCES

(1) Riande, E.; Diaz-Calleja, R.; Prolongo, M. G.; Masegosa, R. M. Polymer Viscoelasticity. Stress and Strain in Practice; Marcel Dekker, Inc.: New York, Basel, 2000, p 879.

(2) Navaruckiene, A.; Bridziuviene, D.; Raudoniene, V.; Rainosalo, E.; Ostrauskaite, J. Vanillin acrylate-based thermo-responsive shape memory antimicrobial photopolymers. *Express Polym Lett* **2022**, *16*, 279–295. DOI: 10.3144/expresspolymlett.2022.22
